# High tumor mutational burden predicts worse prognosis for cervical cancer treated with radiotherapy

**DOI:** 10.1007/s11604-021-01230-5

**Published:** 2021-12-03

**Authors:** Norichika Ota, Yuya Yoshimoto, Narisa Dewi Maulany Darwis, Hiro Sato, Ken Ando, Takahiro Oike, Tatsuya Ohno

**Affiliations:** 1 Department of Radiation Oncology, Gunma University Graduate School of Medicine, 3-39-22, Showa-machi, Maebashi, Gunma 371-8511 Japan; 2 Department of Radiation Oncology, School of Medicine, Fukushima Medical University, 1, Hikarigaoka, Fukushima, 960-1295 Japan; 3 Department of Radiation Oncology, Faculty of Medicine Universitas Indonesia, Dr. Cipto Mangunkusumo National General Hospital, Jl. Diponegoro No. 71, Jakarta Pusat, DKI Jakarta 10430 Indonesia; 4 Gunma University Heavy Ion Medical Center, 3-39-22, Showa-machi, Maebashi, Gunma 371-8511 Japan

**Keywords:** Cervical cancer, Radiotherapy, Tumor mutational burden, Prognosis

## Abstract

**Purpose:**

Tumor mutational burden (TMB) is a surrogate biomarker of neo-antigens and high TMB status is associated with favorable response to immune-checkpoint inhibitors (ICIs). This study aimed to elucidate the association between TMB and the outcome of definitive radiotherapy in patients with cervical cancer.

**Materials and methods:**

TMB and treatment outcome were retrospectively analyzed in patients with newly diagnosed cervical cancer treated with definitive radiotherapy available with somatic mutation data of pre-treatment tumors obtained using a commercially available gene panel.

**Results:**

The study enrolled 98 patients (median follow-up period, 61 months). The median TMB was 9.5 mutations per megabase (range, 3.0–35.5 mutations per megabase). After dichotomization based on this median value, the 5-year overall survival (OS) for TMB-high patients was significantly worse than that of TMB-low patients (61.1% vs. 82.2%). Multivariate analysis identified high TMB status as a significant prognostic factor for worse OS, along with advanced stage, para-aortic lymph node involvement, and absence of concurrent chemotherapy.

**Conclusion:**

These data indicate that TMB is a potential prognostic factor for worse survival in patients with cervical cancer treated with definitive radiotherapy, thereby providing a rationale for treatment of TMB-high cervical cancers with a combination of ICIs plus radiotherapy.

**Secondary abstract:**

This retrospective study of 98 patients demonstrates for the first time that tumor mutational burden (TMB) is an independent prognostic factor for worse overall survival of patients treated with definitive radiotherapy, providing a rationale for treatment of TMB-high cervical cancers with a combination of immune-checkpoint inhibitors plus radiotherapy.

## Introduction

Cervical cancer arises in nearly 0.5 million women annually worldwide, and mortality ranks fourth among all cancers [1]. Radiotherapy is the standard definitive treatment for locally advanced cervical cancer [2]. The treatment outcome has been improved dramatically along with the technological advancement in three-dimensional image-guided adaptive brachytherapy [3]. Nevertheless, a subset of patients develops local recurrence or metastasis after definitive radiotherapy, highlighting the need to identify such patients and stratify them to receive treatments with greater intensity. Immune-checkpoint inhibitors (ICIs) are an emerging candidate for use in combination with radiotherapy. A randomized phase 3 PACIFIC study showed that consolidation therapy with an anti-programmed death ligand-1 antibody prolongs survival of patients with locally advanced non-small cell lung cancer treated with chemo-radiotherapy [4]. In addition, a number of clinical trials are underway to test the efficacy of the ICI-radiotherapy combination against various cancers [5].

Accumulating evidence suggests that ionizing radiation (IR) induces antitumor immune responses [5]. When a tumor is irradiated, neo-antigens are released by dying tumor cells [6–8]. These neo-antigens are taken up by antigen-presenting cells, which trigger a T cell-mediated antitumor immune response [8]. Thus, the amount of neo-antigen per cell may determine the strength of the antitumor immune response post-IR. Neo-antigens derive from somatic mutations in tumors [9, 10]. From this standpoint, the tumor mutational burden (TMB), defined as the number of somatic mutations per megabase (mut/Mb) of an interrogated genomic sequence, is believed to be a surrogate biomarker of neo-antigens [11]. From this perspective, there is a possibility that a high TMB is associated with favorable outcomes after radiotherapy due to a stronger antitumor response; by contrast, a high number of mutations may also be associated with a poor outcome. However, the association between TMB and outcome after definitive radiotherapy in patients with cervical cancer remains unclear. To address this issue, we investigated the association of TMB and treatment outcome in retrospectively collected patients with newly diagnosed cervical cancer treated with definitive radiotherapy available with somatic mutation data of pre-treatment tumors obtained using a commercially available gene panel.

## Materials and methods

### Study cohort

Patients who met the following inclusion criteria were enrolled retrospectively: (i) newly diagnosed and pathologically confirmed squamous cell carcinoma, adenocarcinoma, or adenosquamous carcinoma of the cervix; (ii) staged as IB–IVA based on the International Federation of Gynecology and Obstetrics (FIGO) 2008 staging system; (iii) treated with definitive radiotherapy at Gunma University Hospital from 2006 to 2013; and (iv) available somatic mutation data for pre-treatment tumors (see below for details). The study was conducted in accordance with the principles of the Declaration of Helsinki and was approved by the institutional review board of Gunma University Hospital (approval number 1109). The requirement for informed consent was waived by the institutional review board of Gunma University Hospital due to the opt-out design of the study.

### Radiotherapy

Details regarding definitive radiotherapy were described previously [12]. Briefly, radiotherapy comprised external beam radiotherapy (EBRT) and high-dose-rate brachytherapy. For EBRT, 50 Gy was delivered to the whole pelvis in 25 fractions (five fractions per week). Central shielding was used for the last 30 Gy and 20 Gy in patients with lymph node-negative stage I–II squamous cell carcinoma with a tumor diameter ≤ 4 cm and the others, respectively. Boost irradiation (6–10 Gy in 3–5 fractions) was performed for positive nodes.

Brachytherapy was delivered across four sessions (one session per week); 24 Gy was delivered to the D_90_ high-risk clinical target volume (HR-CTV) using an ^192^Ir remote-after-loading system. Fletcher-Suit Asian Pacific applicators were used mainly, and trocar point needles were added for bulky or irregularly shaped tumors with the aim of optimizing dose distribution. Three-dimensional image-guided treatment planning was performed using in-room computed tomography (CT) based on the recommendations of the Groupe Européen de Curiethérapie and the European Society for Radiotherapy and Oncology.

Patients with stage III–IV disease, tumor diameter > 40 mm, non-squamous cell carcinoma, or nodal involvement received weekly cisplatin-based chemotherapy (40 mg/m^2^) concurrently with EBRT.

The first day of radiotherapy was defined as Day 1. Patients were followed up every 1–3 months for the first 2 years post radiotherapy, and then every 3–6 months for the subsequent 3 years. Disease status was assessed at each follow-up by gynecological examination and imaging (CT or magnetic resonance). Overall survival (OS), progression-free survival (PFS), pelvic recurrence-free survival (PRFS), and distant metastasis-free survival (DMFS) were recorded. PRFS was defined as no evidence of primary tumor regrowth or recurrence in the pelvic region.

Tumor mutational burden.

Somatic mutation data for pre-treatment tumors were obtained as previously described [13]. Briefly, tumor tissues were obtained by pre-treatment punch biopsy. Next, formalin-fixed paraffin-embedded (FFPE) specimens were generated and DNA was extracted from specimens containing at least 20% tumor tissue using the QIAamp DNA FFPE Tissue kit (Qiagen, Hilden, Germany). The degree of DNA fragmentation was examined using the TaqMan RNase P Detection Reagents kit (Thermo Fisher Scientific, Waltham, MA, USA). Amplicon libraries were prepared using the Ion AmpliSeq Library Kit 2.0 (Thermo). The nucleotide sequence of 95.4% of the exons of 409 cancer-related genes was determined using the Ion AmpliSeq Comprehensive Cancer Panel (CCP, Thermo) and the Ion Torrent sequencer (Thermo). Sequence data were analyzed using the Ion Torrent systems (Thermo) with the Genome Reference Consortium Human Build 37 (hg19) as a reference. Single-nucleotide polymorphisms (SNPs) were removed using the SNP data for subject NA12878 in the 1000 Genome project as reference. Somatic mutations were identified using the criteria reported previously [13]. Briefly, the following cutoffs were used: total coverage > 20; variant coverage > 10; variant frequency > 15%; minor allele frequency < 0.1%. The dbSNP database was used to exclude SNPs from the called variants. The TMB for each sample was calculated by dividing the number of somatic mutations by 1.688650 megabases, i.e., the total length of the sequence target.

### Statistical analysis

Difference in numerical variables between two groups was examined using the Mann–Whitney U test. The association between categorical variables was examined using Fisher's exact test. The probability of OS, PFS, PRFS, and DMFS was estimated using the Kaplan–Meier method and the results were compared using the log-rank test. These analyses were performed using R (R Foundation for Statistical Computing, Vienna, Austria) on the EZR platform [14]. Univariate and multivariate analyses were performed using Cox proportional hazard regression in STATA (SE13, StataCorp, College Station, TX, USA). Variables showing P value smaller than 0.25 in univariate analysis were included in subsequent multivariate analysis [15]; variables that showed borderline significance were also included when they were considered to be clinically relevant, based on the part experience and common sense nature of the model-building strategy [16]. The proportional hazards assumption of a given multivariate model was tested using estat phtest function of STATA [17]. Final multivariate model was created after excluding the variables rejected by the test. The level of significance was set at *P* < 0.05.

## Results

**Fig. 1:**
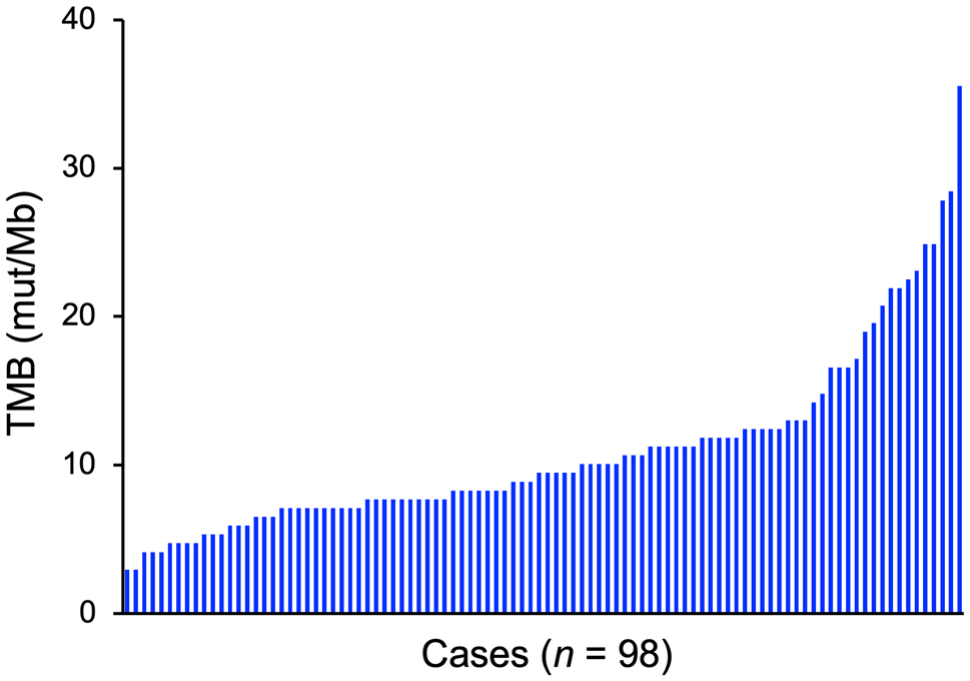
Overview of the tumor mutational burden (TMB) identified in this study cohort. *mut/Mb* mutations per megabase

This study analyzed data from 98 patients. The median follow-up period was 61 months (range, 4–131 months) and the median TMB was 9.5 mut/Mb (range, 3.0–35.5 mut/Mb) (Fig. 1). The study cohort analyzed hereafter was dichotomization based on the median TMB value. The number of TMB-high patients in the squamous cell carcinoma group was significantly higher than that in the other groups (*P* = 0.026). There was no significant difference between the TMB-high and -low patients in terms of follow-up period, age, FIGO stage, tumor diameter, lymph node involvement, and the use of concurrent chemotherapy (Table 1).

**Table 1:** Patient characteristics

Characteristics	All (*n* = 98)	TMB-low (*n* = 53)	TMB-high (*n* = 45)	*P*
Follow-up period (month)	61 (4–131)	61 (4–127)	60 (8–131)	0.40
Age	59 (29–88)	58 (29–88)	60 (34–82)	0.83
FIGO stage				
IB	11 (11.2%)	4 (7.5%)	7 (15.6%)	0.42
II	40 (40.8%)	25 (47.2%)	15 (33.3%)	
III	40 (40.8%)	20 (37.7%)	20 (44.4%)	
IVA	7 (7.1%)	4 (7.6%)	3 (6.7%)	
Tumor diameter				
< 40 mm	15 (15.3%)	10 (18.8%)	5 (11.1%)	0.50
40–60 mm	53 (54.1%)	26 (49.1%)	27 (60.0%)	
> 60 mm	30 (30.6%)	17 (32.1%)	13 (28.9%)	
Pelvic LN involvement				
Negative	47 (48.0%)	28 (52.8%)	19 (42.2%)	0.31
Positive	51 (52.0%)	25 (47.2%)	26 (57.8%)	
PALN involvement				
Negative	85 (86.7%)	45 (84.9%)	40 (88.9%)	0.76
Positive	13 (13.3%)	8 (15.1%)	5 (11.1%)	
Histological type				
SCC	82 (83.7%)	40 (75.5%)	42 (93.3%)	0.026
Others	16 (16.3%)	13 (24.5%)	3 (6.7%)	
Concurrent chemotherapy				
Yes	64 (65.3%)	34 (64.2%)	30 (66.7%)	0.83
No	34 (34.7%)	19 (35.8%)	15 (33.3%)	

*FIGO* the International Federation of Gynecology and Obstetrics 2008, *LN* lymph node, *PALN* para-aortic lymph node, *SCC* squamous cell carcinoma, *TMB* tumor mutational burden. *TMB-low* minimum to median (i.e., 9.5 mutations per megabase). *TMB-high* above median. The follow-up period and age are presented as the median (range) value. Others include 12 adenocarcinomas and four adenosquamous carcinomas. *P* values were calculated from the Mann–Whitney *U* test (follow-up period and age) or Fisher's exact test (other variables)

**Fig. 2:**
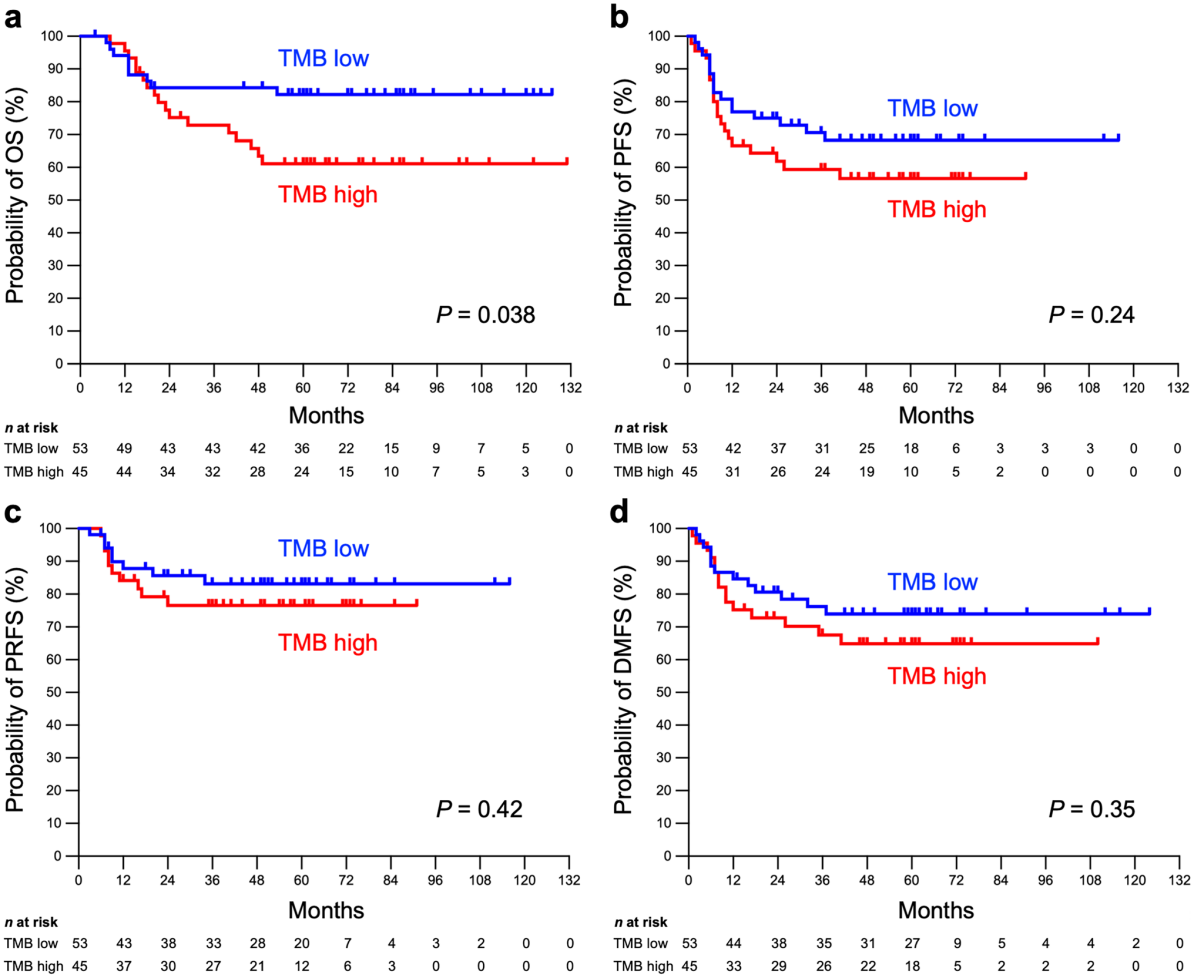
Kaplan–Meier survival estimates stratified by tumor mutational burden (TMB). **a** Overall survival (OS). **b** Progression-free survival (PFS). **c** Pelvic recurrence-free survival (PRFS). **d** Distant metastasis-free survival (DMFS). TMB-low group, minimum to median (i.e., 9.5 mutations per megabase); TMB-high group, above median. *P* values were calculated using the log-rank test

The 5-year OS rate for TMB-high and -low patients was 61.1% and 82.2%, respectively. Interestingly, the OS rate of TMB-high patients was significantly worse than that of TMB-low patients (*P* = 0.038) (Fig. 2a). The PFS, PRFS, and DMFS rates showed similar a trend toward a worse prognosis for TMB-high patients, although they did not reach statistical significance (Fig. 2b–d).

Notably, multivariate analysis identified high TMB status as a significant prognostic factor for worse OS (*P* = 0.024) (Table 2). In addition, FIGO stage IV, the presence of para-aortic lymph node involvement, and the absence of concurrent chemotherapy were significant independent prognostic factors for worse OS (*P* = 0.0030, 0.035, and 0.044, respectively) (Table 2).

**Table 2:** Univariate and multivariate analyses of overall survival

Variables	Univariate		Multivariate	
	HR (95% CI)	*P*	HR (95% CI)	*P*
Age	1.00 (0.97–1.03)	0.89		
FIGO stage				
IB	1		1	
II	0.85 (0.17–4.22)	0.84	1.16 (0.23–5.81)	0.85
III	2.11 (0.48–9.35)	0.32	2.21 (0.46–10.56)	0.32
IVA	7.92 (1.5–41.07)	0.014	12.18 (2.28–65.09)	0.0030
Tumor diameter	1.02 (1.00–1.04)	0.018		
Pelvic LN involvement				
Negative	1			
Positive	1.47 (0.68–3.21)	0.32		
PALN involvement				
Negative	1		1	
Positive	2.84 (1.14–7.08)	0.025	3.04 (1.08–8.56)	0.035
Histological type				
SCC	1			
Others	1.17 (0.44–3.11)	0.75		
Conc. Chemotherapy				
No	1		1	
Yes	0.64 (0.29–1.39)	0.25	0.43 (0.19–0.98)	0.044
TMB				
Low	1		1	
High	2.28 (1.02–5.12)	0.046	2.59 (1.13–5.92)	0.024

*CI* confidence interval, *Conc.* Concurrent, *FIGO* the International Federation of Gynecology and Obstetrics 2008, *HR* hazard ratio, *LN* lymph node, *PALN* para-aortic lymph node, *SCC* squamous cell carcinoma, *TMB* tumor mutational burden. Others include 12 adenocarcinomas and four adenosquamous carcinomas. ref, reference. P values assessed by Cox proportional hazard regression are shown

**Fig. 3:**
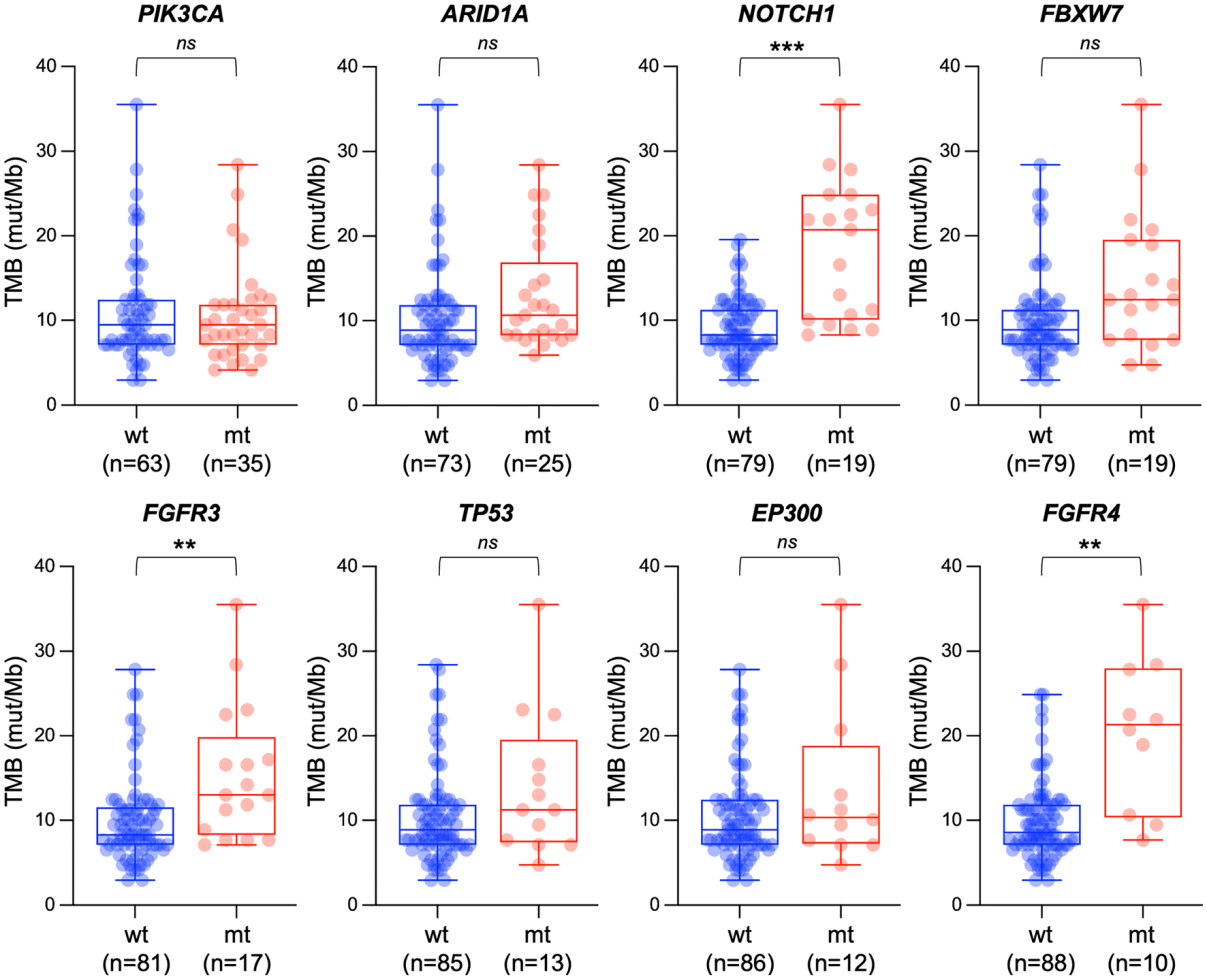
Association between tumor mutational burden (TMB) and mutation status of genes frequently mutated in this cohort (i.e., prevalence > 10%). wt, wild-type; mt, mutant. **P < 0.01; ***P < 0.001, assessed by the Mann–Whitney *U* test after Bonferroni correction. *ns* not significant

Finally, the association between TMB and mutation profile was analyzed for genes that are recurrently mutated in this cohort (i.e., prevalence > 10%) [13]. The TMB of tumors harboring mutations in NOTCH1, FGFR3, and FGFR4 was significantly greater than that for those not harboring these mutations (*P* = 0.0008, 0.0096, and 0.0016, respectively) (Fig. 3). A trend toward a greater TMB was observed for tumors harboring mutations in ARID1A or FBXW7 (*P* = 0.13 and 0.08, respectively). Interestingly, the TMB was highly consistent between PIK3CA-wild-type and -mutant tumors (*P* > 0.99), indicating the potential role of these PIK3CA mutations as drivers.

## Discussion

To the best of our knowledge, this is the first study to demonstrate the prognostic significance of TMB for cervical cancer treated with radiotherapy. Considering that cancers with a high TMB show a favorable response to ICIs [11, 18], the results provide a rationale for testing the combination of ICIs with radiotherapy as a treatment for TMB-high cervical cancers.

Accumulating evidences from various types of cancer suggest that TMB predicts a favorable response to ICIs [11, 18]. However, there is limited and conflicting evidence of an association between TMB and outcome after radiotherapy. Jia et al. investigated patients with non-small cell lung cancer registered in The Cancer Genome Atlas (TCGA) and found that in 117 patients treated with radiotherapy, survival of the TMB-high group was better than that for the TMB-low group; however, TMB had no prognostic significance in 738 patients not treated with radiotherapy [19]. These results are reasonable given that the tumors with a higher TMB release greater amounts of neo-antigens, which may trigger stronger antitumor immune responses. However, we observed the opposite results in the present study, i.e., high TMB status correlated with worse OS in patients with cervical cancer treated with definitive radiotherapy. Similar to our study, Yuan et al. examined 18 patients with esophageal cancer treated with radiotherapy and reported a trend toward worse OS for TMB-high group [20]. These results indicate that the effect of TMB on radiotherapy outcome may differ according to cancer type and the relevant tumor microenvironment. Although the molecular mechanisms underlying radio-resistance of high TMB tumors remain unclear, Jang et al. reported the results of an analysis of single-cell RNA sequencing dataset for breast cancer, showing that radio-resistant cells were enriched with high TMB, high PD-L1 expression, and upregulated Nrf2 pathway [21]. Further research is needed to elucidate the underlying biological mechanisms. In addition, the current study could not determine whether high TMB status contributed to poor survival due to poor pelvic control or due to a high probability of distant metastasis (Fig. 2c,d). The effect of TMB on the pattern of recurrence or metastasis and the relevant molecular mechanisms are unknown, warranting further research.

TMB is measured accurately by whole exome sequencing (WES); however, this approach is impractical in the clinic due to the high cost of sequencing such large genomic regions (i.e., > 30 Mb). Instead, target-capture sequencing using a gene panel is commonplace in clinical practice [22]. Previous in silico studies based on public WES data show high concordance between gene panel-based TMB and WES-based TMB [22, 23]. Another study shows that the accuracy of gene panel-based TMB is influenced by the length of the interrogated sequence. Garofalo et al. reported that the accuracy of gene panel-based TMB decreases when the sequence length is less than 0.5 Mb [24]. Buchhalter et al. reported that 1.5–3 Mb is the best sequence length to estimate TMB using gene panels, and that shorter sequence lengths would lead to overestimation of the TMB [25]. The length of the gene panel used in this study (i.e., CCP by Thermo) is approximately 1.6 Mb; importantly, Hatakeyama et al. reported that the CCP-based TMB of 2040 tumors showed high concordance with WES-based TMB (correlation coefficient, 0.96) [26]. Taken together, these data suggest that the choice of gene panel in this study was robust in terms of TMB measurement. Nevertheless, TMB is also influenced by inter-lab differences in various parameters, including the type and quality of specimens, the kits used for DNA extraction and library preparation, and pipelines used for quality filtering and mutation calling. Thus, further standardization is needed for clinical application of TMB measurement.

In this study, TMB-high status was more common in squamous cell carcinoma, as well as in tumors harboring mutations in NOTCH1, FGFR3, or FGFR4. To date, no clear association between TMB and histology has been reported [11, 18]. In addition, no previous studies have reported enrichment of NOTCH1, FGFR3, or FGFR4 among TMB-high tumors. Thus, these results warrant further validation.

This study has the following limitations. First, the present cohort is highly heterogeneous in terms of patient background (e.g., stage, the presence or absence of concurrent chemotherapy, and tumor size) considering the cohort size. Second, the present cohort lacked DNA extracted from normal tissues. Therefore, removal of SNPs might be insufficient and may introduce potential bias and lead to increased TMB values. In fact, the median TMB in this study (i.e., 9.5 mut/Mb) was relatively higher than that reported in previous studies; for example, Sha et al. reported that the median TMB in TCGA-registered cervical cancers was approximately 5 mut/Mb [11], whereas Shao et al. reported that the median TMB in 114 cervical cancers was 4.4 mut/Mb [27]. Further studies employing a greater number of participants with more homogeneous background, available with matched tumor-normal tissue pairs, are needed.

In summary, this retrospective analysis of data from a commercially available gene panel demonstrates for the first time that TMB is a potential prognostic factor for worse survival of patients with cervical cancer treated with definitive radiotherapy. These data provide a rationale for testing the combination of ICIs plus radiotherapy as a treatment for TMB-high cervical cancers.
